# Awareness and Knowledge of the Adverse Effects of Dermal Fillers Among the Saudi Population: A Cross-Sectional Study

**DOI:** 10.7759/cureus.40322

**Published:** 2023-06-12

**Authors:** Yousef M Al Mashhrawi, Taif F AlNojaidi, Raghad A Alkhaldi, Naif S Alshami, Abdulmajeed S Alhadlaq

**Affiliations:** 1 College of Medicine, Imam Mohammad Ibn Saud Islamic University, Riyadh, SAU; 2 College of Medicine, Al Maarefa University, Riyadh, SAU; 3 Department of Plastic Surgery, Imam Mohammad Ibn Saud Islamic University, Riyadh, SAU

**Keywords:** aesthetic procedures, hyaluronic acid, fillers, awareness, adverse effects

## Abstract

Introduction

Dermal filling for aesthetics and facial rejuvenation is one of the most common aesthetic procedures, with hyaluronic acid (HA) being the most commonly used filler due to its high safety profile. Nevertheless, adverse effects have been reported that people should be aware of before the procedure. This study aims to assess Saudi Arabians' awareness of dermal fillers, their side effects, and information sources.

Methods

This cross-sectional study used an online questionnaire to determine participants’ level of knowledge, from January 2, 2022, to February 19, 2023. Statistical analysis and ordinal logistic regression were used to assess the respondents’ knowledge of dermal fillers and other parameters.

Results

Of the 1,208 respondents, 290 reported using fillers, and 44% reported that complications were mentioned to them before the procedure. The results also showed that the primary source of information was social media (44.8%), followed by the Internet, physicians, and books. Respondents to whom physicians explained complications reported bruising, bluish discoloration at the injection site, redness, swelling, and accumulation of body cells under the skin, as discussed with them. Bruising and bluish discoloration were the significantly reported complications in people who received fillers, whereas lumps, epistaxis, ulceration/loss of skin, and swelling/accumulation were reported in those who did not receive fillers.

Conclusion

Although some adverse effects may occur with HA, its enormous advantages led to its use in facial rejuvenation. With social media as a major source of information, many respondents reported unlikely adverse effects as common ones, suggesting that information sources need to be corrected by physicians to raise awareness of the adverse effects of cosmetic procedures and enhance informed decision-making by patients.

## Introduction

Procedures for aesthetic purposes have gained popularity recently and have become well-accepted [1]. According to the International Society of Aesthetic Plastic Surgery 2021 statistics, dermal fillers are the second most popular non-surgical procedure after onabotulinum toxin A, accounting for 30% of all non-surgical operations [2].

Dermal fillers are advantageous in aesthetics and facial rejuvenation; they give immediate results with a short downtime and are less time-consuming [3,4]. Moreover, hyaluronic acid (HA) has been shown to have a high safety profile due to the presence of the “hyaluronidase” enzyme, which aids in dissolving the substance itself (hyaluronic acid) and reversing the effect of HA to prevent complications [3,5]. The following are some elements influencing its attractiveness. HA is the most widely used dermal filler [6,7]. It acts by maintaining hydration in the dermis, as it is made of glycosaminoglycan, the main component of the extracellular matrix [6]. It also exhibits natural non-immunogenic properties, anti-inflammatory activity, and tissue regeneration [8]. The enormous advantages of HA make it a valuable component in cosmetic procedures such as restoring lip fullness and minimizing wrinkles. Although HA has been proven to have a significant safety margin and efficiency and has become the most widely used dermal filler, there have been unfavorable reactions that, if neglected, can result in serious complications [8,9]. Bruising, erythema, swelling, granuloma formation, pain, and delayed hypersensitivity are possible adverse effects [10]. Certain drugs, such as anticoagulants and herbal supplements, can contribute to bruising [10]. Molecularly, HA is composed of a natural sugar complex that binds to water and causes swelling in certain targeted areas, which is known to be the mechanism of action [10]. Patients should be aware of pain and discomfort during injection and informed that numbing creams only minimize pain [10]. However, significant discomfort is an abnormal sign and may be caused by an allergic response or abscess formation, which may occur after injection [10]. Skin necrosis caused by an intravascular embolism or extravascular compression is a serious complication that should be addressed quickly [10]. Patients should be informed and aware of the signs of skin ischemia and should seek prompt treatment to prevent necrosis [10]. Delayed hypersensitivity reactions to fillers, such as refractory and visceral angioedema, can manifest many months after the procedure [11]. Nevertheless, the hypersensitivity reaction could also result from hyaluronidase as an immediate or delayed reaction [12]. Hence, before starting any procedure, patients should be informed of the potential side effects and complications and taught about the risks by their physicians [13]. During consultations, physicians should obtain a history from patients, as some patients may take certain drugs that interact with the dermal fillers, or for the physician to be aware of any patient allergies that may cause adverse effects such as the type of filler or onabotulinum toxin A; similarly, consent should be obtained from the patient [13]. Furthermore, physicians should be proficient in the technique used to augment lips with HA fillers to promote patient safety and satisfaction and avoid overall complications [14]. Patients’ adequate counseling can aid in the prevention of undesirable effects and improve results [13,15].

This study aimed to determine the proportion of individuals informed about the adverse effects of dermal fillers in the clinic before treatment and assess public knowledge about the associated risks. Using a Google survey, we aimed to investigate people’s knowledge of dermal fillers and assess how trustworthy the sources of information are that led to their awareness of its detrimental effects, as well as to evaluate physicians’ participation in changing people’s perceptions about soft tissue fillers and their harmful effects. Social media have increasingly replaced other information sources for individuals seeking guidance, specifically regarding their health. The study also aims to examine the reliability and integrity of social media in terms of dermal fillers and their adverse consequences, as well as physician-patient communication and whether the adverse effects and problems were disclosed to patients before treatment.

## Materials and methods

Study design

This cross-sectional study was approved by the Institutional Review Board of Imam Mohammad Ibn Saud Islamic University (IRB No. 183-2021) and performed in accordance with the principles of the Declaration of Helsinki. Informed consent was waived. A Google survey was used as a self-reported anonymous questionnaire distributed across social media platforms to participants from Saudi Arabia. The study’s questionnaire timeline was between January 2, 2022, and February 19, 2023. The inclusion criteria were participants aged ≥18 years who lived in Saudi Arabia. The exclusion criteria were participants aged <18 years and living outside Saudi Arabia. All the enrolled participants met the inclusion criteria and were included in the analysis. Participants were provided with a brief explanation of the study topic for reading and understanding. The survey began with demographic questions, followed by questions on whether the participants have had filler injections and whether their physician explained the common potential adverse effects associated with fillers; if the answers were yes, they had to specify what they were. The survey also assessed participants’ knowledge level regarding dermal fillers and their source of information about the topic because the study participants were people who have or have not had fillers; thus, we intended to compare if there was a difference in their knowledge level about the adverse effects. Using Raosoft.com, the study’s sample size recommended was 377 participants with a margin of error of 5% and a 95% confidence level. However, the total number of our study respondents was 1,208.

Statistical analysis

Statistical analyses were performed using R version 3.6.3. Categorical variables were summarized as counts and percentages, and continuous normal variables as mean ± standard deviation. In cases of non-normality, the median and interquartile range were used. Hypothesis testing was performed using the unpaired t-test and Mann-Whitney test. The association between categorical variables was assessed using the chi-square test of independence. Regression analysis was used to assess the factors associated with filler use. Age, sex, and education were the independent variables in the model. Ordinal logistic regression was used to assess factors associated with knowledge of fillers. Sex, age, education, previous use, and source of information were used as independent variables. Hypothesis testing was performed at the 5% significance level.

## Results

The study included 1,208 respondents (1020 females and 188 males). Most respondents (80.4%) were aged 18-29 years. Approximately three-quarters of the respondents (73.5%) had a bachelor’s degree, and only 4.72% had a postgraduate degree. More females completed a bachelor’s degree, whereas more males had a postgraduate degree or completed only high school. One-quarter of the respondents reported using fillers (24%, n=290), and this percentage was higher in females than in males. A total of 290 respondents reported using fillers, and 44.8% reported that potential complications of filler injections were explained to them before the procedure. Self-reported knowledge regarding fillers was higher in females, as shown by the higher percentage of females who reported having good and excellent knowledge (31.4% and 14.2%, respectively) than in males (17.6% and 7.14%, respectively) (Table 1).

**Table 1:** Factors associated with filler use Data were summarized using counts and percentages, and analysis was performed using the chi-square test of independence.

	Ever had a filler injection		
Predictors	Odds Ratios	CI	p
(Intercept)	0.20	0.14 – 0.28	<0.001
Gender			
Female	Reference		
Male	0.13	0.06 – 0.24	<0.001
Age			
18-29	Reference		
30-39	2.25	1.40 – 3.58	0.001
40-49	2.43	1.51 – 3.86	<0.001
> 50	1.92	0.96 – 3.72	0.058
Education			
High school or less	Reference		
University	1.62	1.12 – 2.40	0.013
Post-graduate	4.61	2.27 – 9.52	<0.001
Observations	1208		
R^2^ Tjur	0.087		

**Figure 1:**
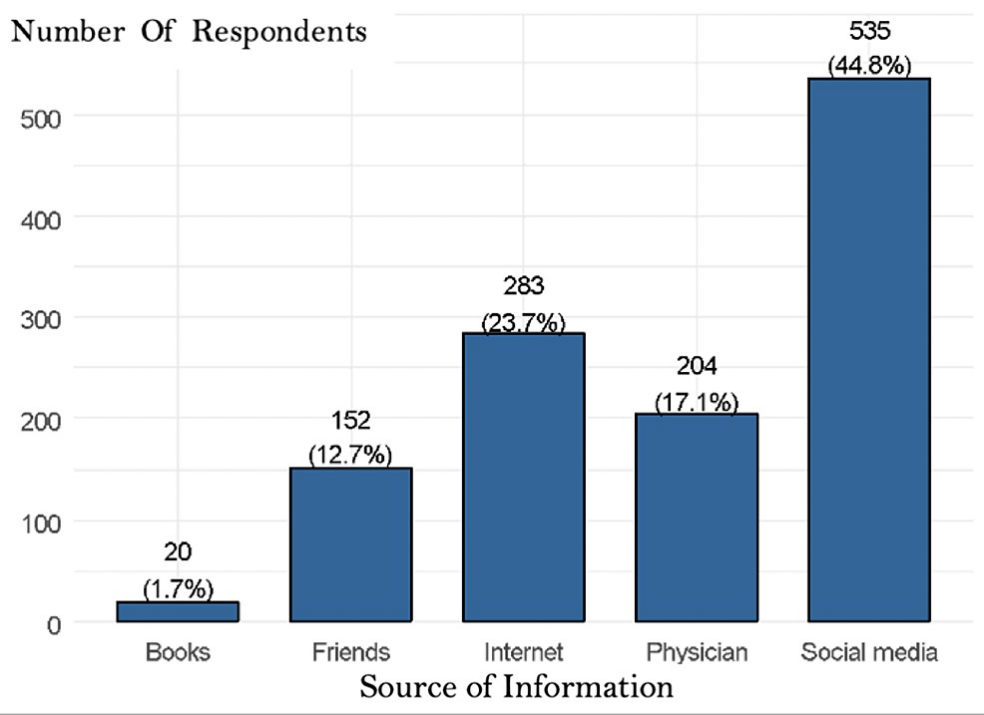
The primary source of information regarding filler

The results showed that social media (44.8%) was the primary source of information for respondents, followed by the internet (23.7%), physicians (17.1%), and friends (12.7%) (Figure 1).

**Figure 2:**
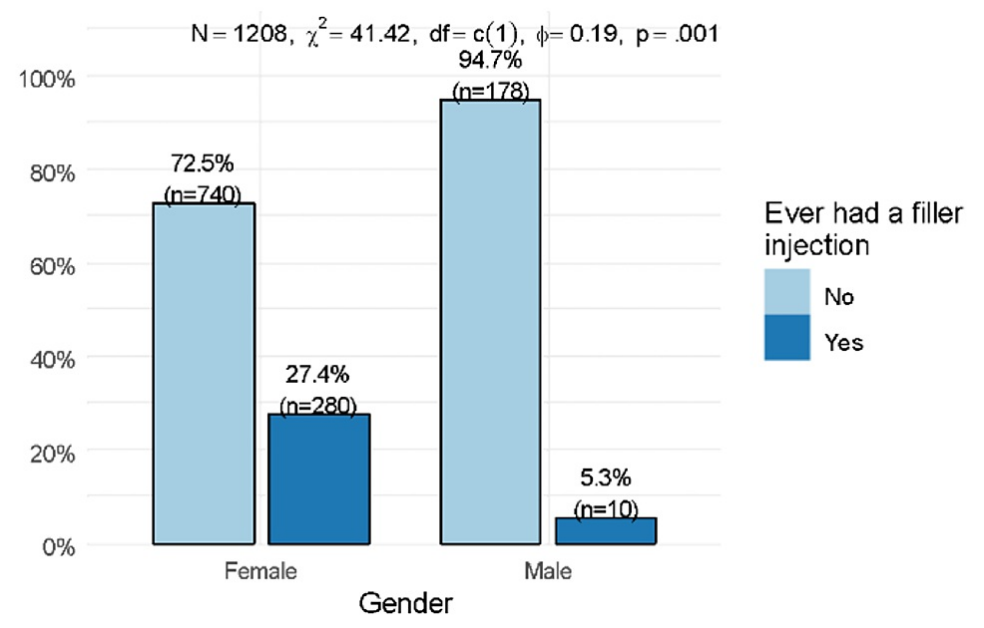
Association between gender and the use of fillers

The analysis of our results showed that females were more likely to use fillers than males (27.4% vs. 5.3%, p<0.001) (Figure 2).

**Figure 3:**
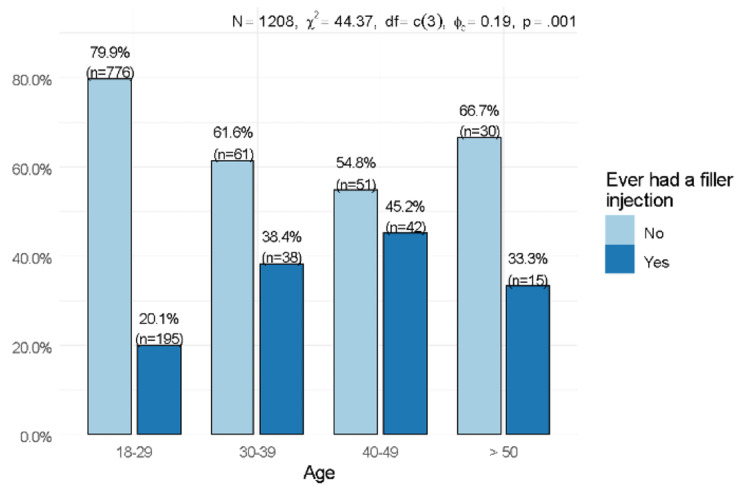
Association between age and the use of fillers

A statistically significant trend was observed in the use of fillers across age groups (p=0.001). The percentage of respondents who used fillers increased from 20.1% among respondents aged 18-29 years to 38.4% and 45.2% among respondents aged 30-39 and 40-49 years, respectively. Only 45 respondents were aged >50 years, which was too small to allow an accurate assessment of filler use in that age group (Figure 3).

Multivariate regression analysis was used to assess demographic factors associated with filler use. Females were more likely than males to report using fillers (OR: 0.13, p<0.001), indicating that the odds of reporting filler use were 87% lower in males than females. The odds of using fillers were higher among older respondents than among younger respondents. In particular, the odds of using fillers were 2.25 and 2.43 times higher in respondents aged 30-39 and 40-49 years, respectively, than in those aged 18-29 years. The odds of filler use were 62% higher in respondents with a university education than in those who had completed only high school (OR: 1.62, p=0.013). The odds were also higher for respondents with postgraduate degrees (OR: 4.61, p<0.001) (Table 2).

**Table 2:** Factors associated with filler use

	Ever had a filler injection		
Predictors	Odds Ratios	CI	P
(Intercept)	0.20	0.14–0.28	<0.001
Gender			
Female	Reference		
Male	0.13	0.06–0.24	<0.001
Age			
18–29	Reference		
30–39	2.25	1.40–3.58	0.001
40–49	2.43	1.51–3.86	<0.001
>50	1.92	0.96–3.72	0.058
Education			
High school or less	Reference		
University	1.62	1.12–2.40	0.013
Postgraduate	4.61	2.27–9.52	<0.001
Observations	1208		
R^2^ Tjur	0.087		

**Figure 4:**
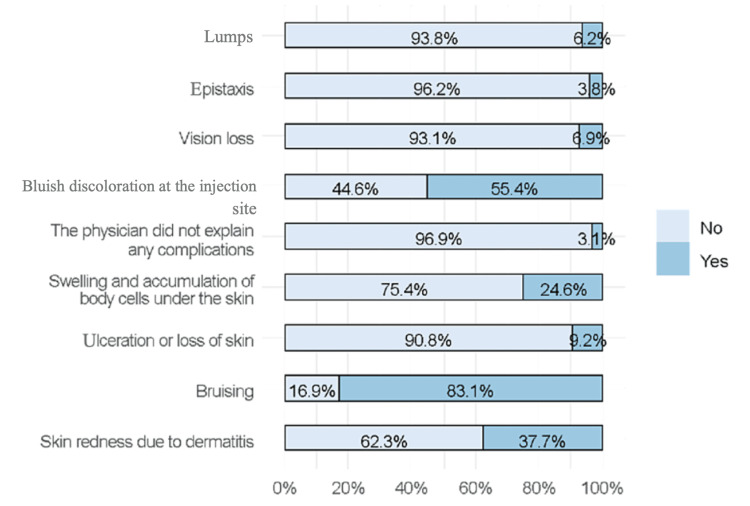
Knowledge of complications reported by respondents (n=130)

From a total of 130 respondents, the majority (96.9%) reported that complications were explained to them by their physicians. The most commonly reported complications were bruising (83.1%), bluish discoloration at the injection site (55.4%), and skin redness due to dermatitis (37.7%). Other reported complications included swelling and accumulation of cells under the skin (24.6%). Only 3.1% mentioned that the physician did not discuss complications with them (Figure 4).

The results showed that participants who did not use fillers reported lumps, epistaxis, ulceration/loss of the skin, and swelling/accumulation of body cells under the skin as possible complications of filler injection. However, bruising and bluish discoloration of the skin were more likely to be reported by respondents who used fillers. The frequency of vision loss and skin redness due to dermatitis was not significantly different between the groups (Table 3).

**Table 3:** Comparison of awareness of complications based on the use of filler Data were summarized using counts and percentages. Analysis was performed using the chi-square test of independence.

Filler Injection	[ALL]	No	Yes	p.overall
	N=1042	N=755	N=287	
Swelling and accumulation of body cells under the skin:				<0.001
No	759 (72.8%)	522 (69.1%)	237 (82.6%)	
Yes	283 (27.2%)	233 (30.9%)	50 (17.4%)	
Skin redness due to dermatitis:				0.283
No	735 (70.5%)	525 (69.5%)	210 (73.2%)	
Yes	307 (29.5%)	230 (30.5%)	77 (26.8%)	
Bruising:				<0.001
No	539 (51.7%)	441 (58.4%)	98 (34.1%)	
Yes	503 (48.3%)	314 (41.6%)	189 (65.9%)	
Ulceration or loss of skin:				0.004
No	945 (90.7%)	672 (89.0%)	273 (95.1%)	
Yes	97 (9.31%)	83 (11.0%)	14 (4.88%)	
Bluish discoloration at the injection site:				<0.001
No	716 (68.7%)	548 (72.6%)	168 (58.5%)	
Yes	326 (31.3%)	207 (27.4%)	119 (41.5%)	
Lumps:				<0.001
No	939 (90.1%)	663 (87.8%)	276 (96.2%)	
Yes	103 (9.88%)	92 (12.2%)	11 (3.83%)	
Epistaxis:				<0.001
No	967 (92.8%)	687 (91.0%)	280 (97.6%)	
Yes	75 (7.20%)	68 (9.01%)	7 (2.44%)	
Vision loss:				0.154
No	983 (94.3%)	707 (93.6%)	276 (96.2%)	
Yes	59 (5.66%)	48 (6.36%)	11 (3.83%)	

Results showed that self-reported knowledge was significantly lower in males than in females (OR: 0.41, p<0.001). The older age groups had less knowledge regarding fillers than the younger age groups (OR: 0.54, p=0.004, and OR: 0.45, p=0.008 for age groups 40-49 and >50, respectively). Education level was not associated with self-reported knowledge. Prior use of fillers was associated with higher odds of having higher knowledge grades (OR: 2.18, p<0.001). Sources of information also showed a statistically significant association with knowledge of fillers. The odds of having more knowledge were higher among respondents who used books as the primary source of information (OR: 5.02, p<0.001). The same was observed for respondents who mentioned physicians as their primary source of information (OR: 2.15, p<00.001) (Table 4).

**Table 4:** Factors associated with knowledge regarding fillers Analysis was performed using ordinal logistic regression.

Predictors	Odds Ratios	CI	p
Gender: Male vs. Female	0.41	0.30 – 0.57	<0.001
Age:			
20 - 29	Ref		
30-39	1.01	0.68 – 1.48	0.976
40-49	0.54	0.36 – 0.82	0.004
>50	0.45	0.25 – 0.81	0.008
Education			
Education: High school	Ref		
Education: University	1.03	0.80 – 1.34	0.802
Education: Post-graduate	0.96	0.54 – 1.71	0.900
Ever had a filler injection: Yes vs. No	2.18	1.67 – 2.83	<0.001
The main source of knowledge about the complication of filler injections			
Social media	Ref		
Books	5.02	2.14 – 11.81	<0.001
Friends	0.61	0.44 – 0.86	0.005
Internet	1.03	0.78 – 1.34	0.851
Physician	2.15	1.58 – 2.92	<0.001

## Discussion

The physician-patient relationship is an essential element in patients’ knowledge and awareness. Positive interactions can help achieve better patient health outcomes. One factor that can help achieve this strong doctor-patient relationship is good communication skills, involving physicians explaining and responding to patients’ concerns and questions. Communication is a major factor in increasing patient awareness and knowledge of cosmetic procedures. Physicians should explain and let the patient know how the procedure is performed, how long it takes, and how to detect any adverse reactions and risks; even though the chances are minimal, they should be addressed. Our findings indicate that social media is the primary source of information for many people. Thus, a significant amount of information people obtain is through social media, which can be misleading or exaggerated for the procedure. Table 3 and Figure 1 showed that when people were asked what they thought the complications of fillers were, those who have not had fillers thought that lumps, epistaxis, ulceration/loss of skin, and swelling/accumulation of body cells under the skin were more common than those who reported bruising and bluish discoloration of the skin (p=0.001). Major complications, such as vision loss and skin redness due to dermatitis, were not significantly different between the two groups. We compared our findings with a narrative review published in the Journal of Cosmetic Dermatology, which revealed that the common adverse effects of injections were erythema, edema, ecchymosis, and pain [15]. It should also be noted that the major responses regarding self-knowledge were mostly good and very good (p<0.001). Additionally, it should be noted that most respondents rated their self-knowledge as mostly good or very good, with significance (p<0.001).

However, based on the discrepancy between what is typically common and what the respondents chose, as pointed out in the research, we can conclude that their knowledge is less accurate, which may be caused by the unreliable or flawed nature of their information sources. Consequently, it can make people more anxious and afraid and prevent them from getting fillers for cosmetic or medical purposes. However, there are also advantages to using social media that draw people into using it as their primary source of information. We found that most respondents were highly educated, especially those who wanted to undergo cosmetic procedures. Therefore, these groups of educated people will mostly obtain their information from a relatively good source, either from the internet or social media, as the results show the primary source of information from social media at around 44.8%, followed by the internet at 23.7%, with books being the least chosen, with only 20 people out of the total. Owing to their simplicity, viability, and time-saving benefits, people frequently turn to the internet and social media as sources. Instead of visiting the clinic, they sought answers online. However, books that were the least chosen, with only 20 out of the 1208 responses, reported using it as their source. This could be due to difficulties obtaining relevant information and the time required to look for specific information. Physicians can handle these responses about major dependency on the internet by correcting people’s misconceptions and spreading accurate information. Hence, physicians should be more active on social media to increase public awareness of cosmetic procedures. They should also update their medical websites with new information on every filler, as we believe this is where the current and upcoming generations will first turn when seeking information. Particularly in this day and age, where social media and the internet have become convenient ways to help people lean more towards it, this input from physicians will help raise people’s awareness. Some limitations of the study include the fact that it was a cross-sectional study that measured people’s awareness level concerning the adverse effects of dermal filler; hence, it is considered a snapshot of patients’ knowledge and perception in a certain period, which does not represent the whole timeline. The outcome presented a need to improve awareness and correct misconceptions regarding dermal fillers and cosmetic procedures. The study sample size was representative; however, because it was a non-probability sample method, social media was the mean of the distribution. It only represents people who have access to the internet and use social media. Another potential limitation of the study is that it relied on self-reported data, which may be subject to recall bias and social desirability bias. Respondents may have over-reported their knowledge level or under-reported their experiences with adverse effects.

## Conclusions

This study examines patients' understanding of dermal fillers and explores the role that a physician plays in educating the public about the possible risks associated with aesthetic procedures. The information’s primary source for responses is social media; therefore, it requires supervision to have accurate facts and prevent misconceptions.

## References

[1] Safran T, Swift A, Cotofana S, Nikolis A (2021). Evaluating safety in hyaluronic acid lip injections. Expert Opin Drug Saf.

[2] Internationally Society of Aesthetic Plastic Surgery (2023). International Society of Aesthetic Plastic Surgery. International survey on aesthetic/cosmetic procedures. ISAPS [Internet.

[3] Walker L, King M (2018). This month’s guideline: visual loss secondary to cosmetic filler injection. J Clin Aesthet Dermatol.

[4] Stojanovič L, Majdič N (2019). Effectiveness and safety of hyaluronic acid fillers used to enhance overall lip fullness: a systematic review of clinical studies. J Cosmet Dermatol.

[5] Cavallini M, Gazzola R, Metalla M, Vaienti L (2013). The role of hyaluronidase in the treatment of complications from hyaluronic acid dermal fillers. Aesthet Surg J.

[6] Joganathan V, Shah-Desai S (2020). Awareness of management of hyaluronic acid induced visual loss: a British National Survey. Eye (Lond).

[7] Doerfler L, Hanke CW (2019). Arterial occlusion and necrosis following hyaluronic acid injection and a review of the literature. J Drugs Dermatol.

[8] Czumbel LM, Farkasdi S, Gede N (2021). Hyaluronic acid is an effective dermal filler for lip augmentation: a meta-analysis. Front Surg.

[9] Jung H (2020). Hyaluronidase: an overview of its properties, applications, and side effects. Arch Plast Surg.

[10] Winslow CP (2009). The management of dermal filler complications. Facial Plast Surg.

[11] Alawami AZ, Tannous Z (2021). Late onset hypersensitivity reaction to hyaluronic acid dermal fillers manifesting as cutaneous and visceral angioedema. J Cosmet Dermatol.

[12] Bass LS (2015). Injectable filler techniques for facial rejuvenation, volumization, and augmentation. Facial Plast Surg Clin North Am.

[13] De Boulle K, Heydenrych I (2015). Patient factors influencing dermal filler complications: prevention, assessment, and treatment. Clin Cosmet Investig Dermatol.

[14] Walker L, Cetto R (2021). Lip augmentation using hyaluronic acid filler and a 4-mm needle: a safer, more natural, and predictable approach. J Clin Aesthet Dermatol.

[15] Kassir M, Gupta M, Galadari H (2020). Complications of botulinum toxin and fillers: a narrative review. J Cosmet Dermatol.

